# Development of an Immunochromatographic Strip Test for the Rapid Detection of Alternariol Monomethyl Ether in Fruit

**DOI:** 10.3390/toxins9050152

**Published:** 2017-04-29

**Authors:** Yan Man, Gang Liang, Fuchao Jia, An Li, Hailong Fu, Meng Wang, Ligang Pan

**Affiliations:** 1 Beijing Research Center for Agricultural Standards and Testing, Beijing Academy of Agriculture and Forestry Sciences, Beijing 100097, China; manyan3669@163.com (Y.M.); liangg@brcast.org.cn (G.L.); lia@brcast.org.cn (A.L.); fuhl@brcast.org.cn (H.F.); wangm@brcast.org.cn (M.W.); 2 Risk Assessment Lab for Agro-Products (Beijing), Ministry of Agriculture P.R. China, Beijing 100125, China; 3 Beijing Municipal Key Laboratory of Agriculture Environment Monitoring, Beijing 100097, China; 4 School of Science, Shandong University of Technology, Zibo 255000, Shandong, China; jiafuchao@sdut.edu.cn

**Keywords:** alternariol monomethyl ether (AME), monoclonal antibody, immunochromatographic strip test, colloidal gold nanoparticle, competitive ELISA

## Abstract

A rapid, portable, and semi-quantitative immunochromatographic strip was developed for rapid and visual detection of alternariol monomethyl ether (AME). For this purpose, the anti-AME monoclonal antibody (mAb) was prepared and identified. AME coupled to bovine serum albumin (BSA) via methyl 4-bromobutanoate was prepared as immunogen. The recoveries of AME in spiked cherry and orange fruits determined by competitive ELISA were 86.1% and 80.7%, respectively. A colloidal gold nanoparticle (CGN) and CGNs-mAb conjugate were synthesized, and on this basis, a competitive colloidal gold immunochromatographic strip was developed and applied to the detection of AME toxin in fruit samples. The intensity of red density of the test line (T line) is inversely proportional to AME concentration in the range 0.1–10 ng/mL. The visual limit of detection (LOD) of AME was found to be about 10 ng/mL. The semi-quantitative detection can be completed in 10 min. Moreover, the immunochromatographic strip has lower cross-reactivity with AME analogues, and it has a good stability performance (following 3 months of storage). Hence, the colloidal gold immunochromatographic strip could be used as a semi-quantitative tool for the on-site, rapid, and visual detection of AME in fruit.

## 1. Introduction

Alternariol monomethyl ether (AME, 3, 7-Dihydroxy-9-methoxy-1-methyl-6H-dibenzo [b, d] pyran-6-one) is one of the major *Alternaria* mycotoxins. AME is structurally related to alternariol (AOH) and altenuene (ALT), and belongs to dibenzo-α-pyrone derivatives which are produced by *Alternaria alternata*, *Alternaria solani* and some other species within the genus *Alternaria* [1,2]. AME is distributed all over the world and shows no acute toxic effects to the health of humans and animals [3], but it possesses the properties of mutagenicity and carcinogenicity, causing particularly high incidences of esophageal cancer [4]. It also can induce DNA breaks [5]. Moreover, AME was characterized as a powerful inhibitor for topoisomerase I and topoisomerase II, contributing to its genotoxicity [6,7]. As a result, it is important and urgent to develop sensitive and specific analytical methods for qualitative and quantitative detection of AME.

At present, the main analytical methods for detecting AME in foods and agricultural products are gas chromatography (GC) and liquid chromatography-mass spectrometry (LC-MS). GC is suitable for detecting non-polar and volatile compounds, however, AME toxin is made up of small, polar, and non-volatile molecules [8], hence, its analysis usually needs derivatization prior to GC analysis [9]. The derivatization results in potential disadvantages for GC analysis such as matrix interference, the time-consuming nature of the process, the need for an expensive derivatization reagent, and so on. In the last few years, LC-MS, especially LC tandem mass spectrometry (LC-MS/MS), has played an important role in AME detection without derivatization [10,11,12]. However, these methods need highly qualified technicians and can only be carried out in the lab.

The colloidal gold immunochromatographic strip test has been developed into a popular platform for application in food, feed, and agricultural products safety due to its potential for rapid, portable, on-site, and visual detection. Until now, it has been used for the detection of small molecular mycotoxins [13], such as ochratoxin A [14,15,16], deoxynivalenol [17,18], aflatoxin B1 [19], aflatoxin M1 [20], fumonisins B1, B2, and B3 [21], zearalenone [22], amatoxins [23], and T-2 Toxin [24,25]. In the current study, a highly specific monoclonal antibody (mAb) against AME was produced using AME- bovine serum albumin (BSA) as immunogen for the development of an anti-AME mAb-based colloidal gold immunochromatographic strip test method. This method was used to detect AME, an *Alternaria* mycotoxin, in fruit samples.

## 2. Results

### 2.1. Carboxyl Derivative Modification of AME

**Figure 1:**
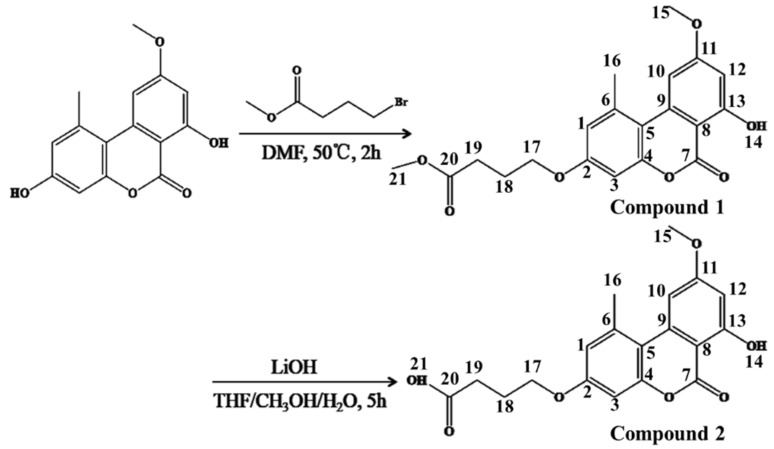
The schematic preparation of carboxyl derivative modification of alternariol monomethyl ether (AME).

The schematic of AME carboxyl derivative modification is shown in Figure 1. AME linked methyl ester (compound **1**) and the AME linked carboxylic acid (compound **2**) were revealed by NMR. The following ^1^H-NMR (400 MHz, DMSO) of AME linked methyl ester (compound **1**) was produced: δ 11.81 (s, 1 H, 14), 7.28 (s, 1 H, 10), 6.91 (s, 2 H, 1 and 3), 6.67 (s, 1 H, 12), 4.10 (t, *J* = 6.1 Hz, 2 H, 17), 3.93 (s, 3 H, 15), 3.63 (s, 3 H, 21), 2.79 (s, 3 H, 16), 2.49–2.42 (m, 2 H, 19), 2.07–1.93 (m, 2 H, 18). The following ^1^H-NMR (400 MHz, DMSO) of AME linked carboxylic acid (compound **2**) was produced: δ 12.17 (s, 1 H, 21), 11.79 (s, 1 H, 14), 7.19 (s, 1 H, 10), 6.85 (d, *J* = 7.0 Hz, 2 H, 1 and 3), 6.61 (d, *J* = 1.7 Hz, 1 H, 12), 4.07 (t, *J* = 6.4 Hz, 2 H, 17), 3.90 (s, 3 H, 15), 2.73 (s, 3 H, 16), 2.41 (t, *J* = 7.3 Hz, 2 H, 19), 1.97 (p, *J* = 6.7 Hz, 2 H, 18). The results of NMR indicated that AME was modified with methyl ester and carboxylic acid successfully, respectively. In addition, the yield and purity were 76% and ˃95% for compound **1**, respectively, and were 28% and >98% for compound **2**, respectively.

### 2.2. Production and Characterization of Anti-AME mAb

**Figure 2:**
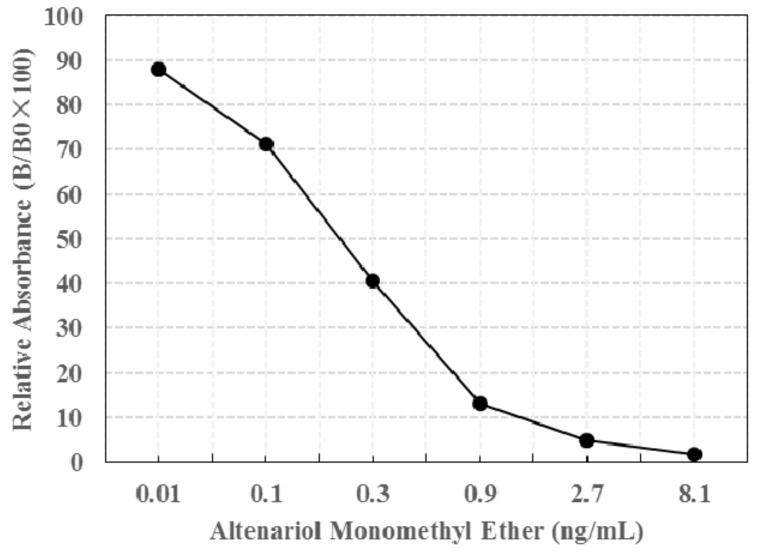
Typical standard curves of the competitive ELISA for alternariol monomethyl ether (AME). Three replicate wells of all the standard concentrations (0.01, 0.1, 0.3, 0.9, 2.7, 8.1 ng/mL) were analyzed.

The specificity and sensitivity of immunoassay developed for detecting mycotoxin are dependent on the characteristics of mAb. Typical standard curves of competitive ELISA for AME are shown in Figure 2. According to Figure 2, the anti-AME mAb displayed a higher affinity for AME, the antibody titer (>1:90,000) was obtained, and the 50% inhibition concentration (IC_50_) values of AME was 0.227 ng/mL. In addition, due to the fact that the chemical structures of AOH and AME are very similar, the cross-reactivity of anti-AME mAb with AOH was also examined using competitive ELISA. The results showed that the anti-AME mAb was specific to AME, whereas the relative weak cross reactivity to AOH was determined to be 2.1%.

### 2.3. Characterization of Colloidal Gold Nanoparticles (CGNs) and the CGNs-mAb Conjugates

**Figure 3:**
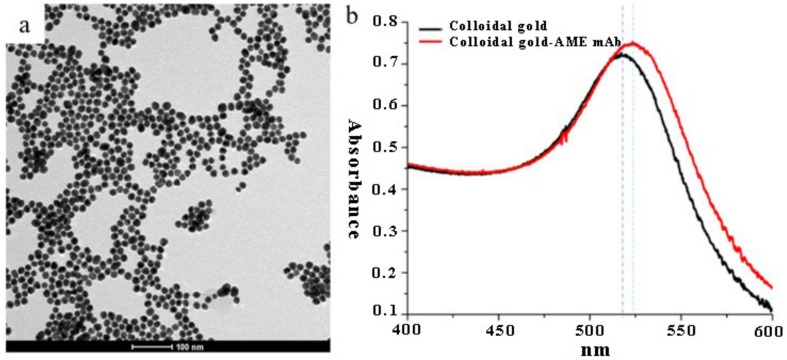
TEM image of colloidal gold nanoparticles (CGNs) (**a**) and UV spectrum of the CGNs-mAb conjugates (**b**). The black line and red line in **b** represent CGNs and CGNs-mAb conjugates, respectively.

A transmission electron microscopy (TEM) image for the CGNs is shown in Figure 3a. The UV spectrum of CGNs and colloidal gold-AME mAb conjugate was detected by UV spectrophotometer at a wavelength of 400 to 600 nm. The CGNs were well dispersed, and the average diameter of the CGNs was about 24 nm. Moreover, the CGNs had a maximum UV absorption wavelength at 517 nm (Figure 3b). When it was combined with anti-AME mAb, the UV absorption wavelength had a red-shift. Here, the UV absorption wavelength of CGNs-mAb conjugates was 524 nm. The results indicated that the CGNs were well prepared, and anti-AME mAb was also successfully conjugated with CGNs.

### 2.4. Assembly and Competitive Detection Principle of Immunochromatographic Strip

**Figure 4:**
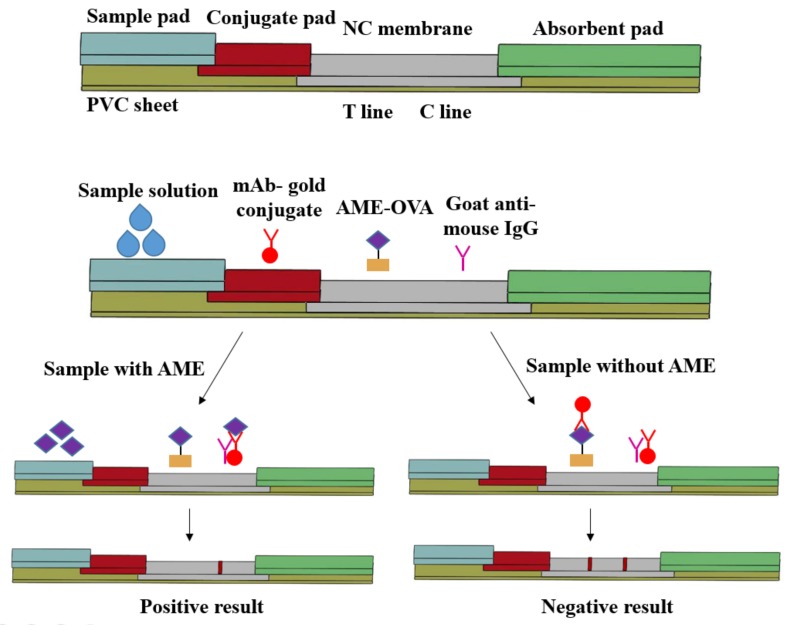
Schematic illustration of immunochromatographic strip. The T line (test line) and C line (control line) was coated by AME- ovalbumin (OVA) and goat anti-mouse IgG, respectively.

An immunochromatographic strip for the on-site detection of AME toxins was developed. Figure 4 shows the format and competitive detection principle. When AME toxins are present in a sample solution, they will be captured by the CGNs-mAb and formed CGNs-mAb-AME conjugates in conjugate pad firstly. The conjugates will be captured by the goat anti-mouse IgG in the C line, whereas they can not be captured by the AME- ovalbumin (OVA) in the T line. As such, for positive results the T line does not show the red color while the C line does show the red color. When the sample solution does not contain AME toxins, the CGNs-mAb conjugates will be captured by AME-OVA and goat anti-mouse IgG in the T line and the C line, respectively. As such, for negative results both the T line and the C line show the red color.

### 2.5. Limit of Detection (LOD) of the Prepared Immunochromatographic Strip

**Figure 5:**
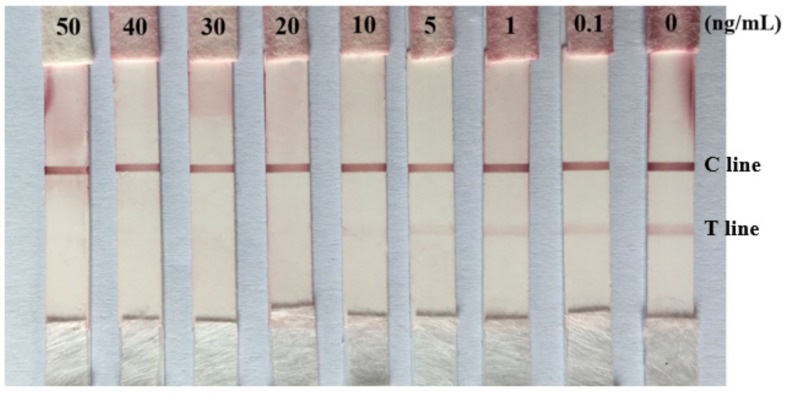
Limit of detection (LOD) of AME with colloidal gold immunochromatographic strip. A series of dilutions (0–50 ng/mL) of AME standard solutions were prepared by diluting AME in PBS. When the concentration of AME was higher than 10 ng/mL, the red line at the T line disappeared.

Several standard solutions of AME at the concentrations of 50, 40, 30, 20, 10, 5, 1, 0.1, and 0 ng/mL were prepared by diluting AME (1 mg/mL in dimethylformamide (DMF)) with PBS. These AME standard solutions (70 μL) were measured by the immunochromatographic strips. The results were judged by visualization within 10 min. As shown in Figure 5, the intensity of red density on T lines reduced when the concentration of AME toxins increased. The color intensity of the colloidal gold immunochromatographic strip tests is inversely proportional to AME concentrations in the range 0.1–10 ng/mL. The visual LOD of AME immunochromatographic strip is defined as the concentration of AME standard solution that causes the T line to be completely invisible. Therefore, the LOD of the strip test for AME was about 10 ng/mL. When the AME concentration was above the LOD, there was not a red line on the T line, and there was only one red line on the C line. On the contrary, if there are two red lines on the nitrocellulose (NC) membrane, it shows that the concentration of AME in sample is below the LOD.

### 2.6. Cross Reactivity Test of the Prepared Immunochromatographic Strip

**Figure 6:**
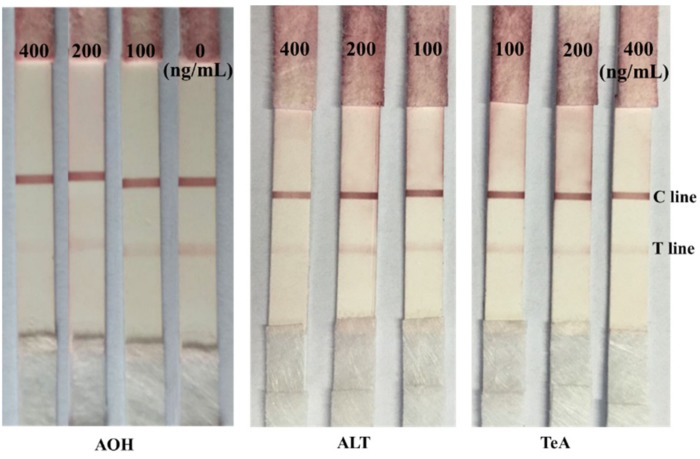
Cross reactivity of the immunochromatographic strip with alternariol (AOH), altenuene (ALT), and tenuazonic acid (TeA).

AOH, ALT, and tenuazonic acid (TeA) have similar structures to AME. They were chosen to study the cross reactivity of the prepared immunochromatographic strips. The concentrations of 400, 200, and 100 ng/mL of AOH, ALT, and TeA were used in the prepared immunochromatographic strips, respectively. As shown in Figure 6, when the concentrations of AOH, ALT, and TeA were at 100, 200, and 400 ng/mL, the intensity of the red color at T lines was slightly weaker compared to that of 0 ng/mL, but the loss of the intensity of red density was not significant. Hence, no significant cross-reactivity was observed with AOH, ALT, and TeA. All of the results indicated that the presence of these mycotoxins (AOH, ALT, and TeA) in the test sample did not interfere with the detection of AME. As such, the prepared immunochromatographic strips have good specificity.

### 2.7. The Stability Performance of the Immunochromatographic Strip for the Detection of AME

**Figure 7:**
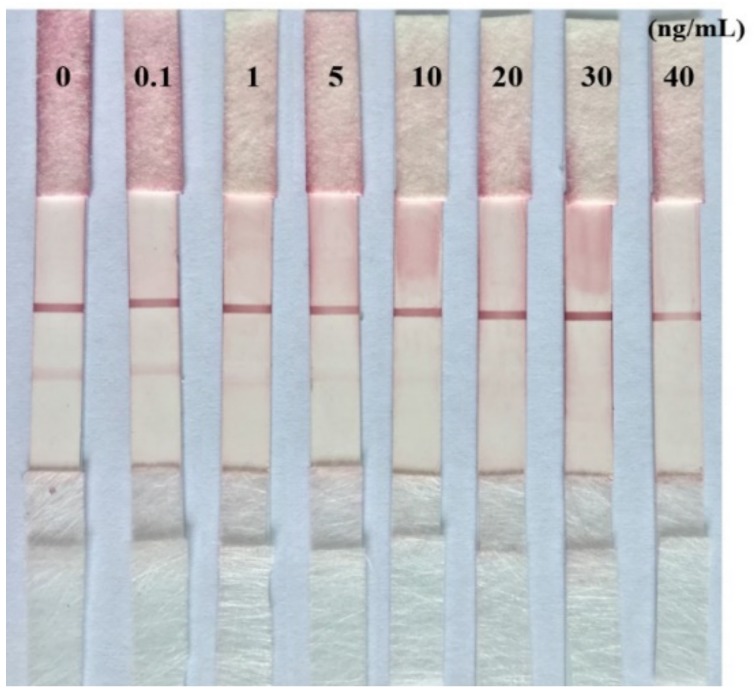
The AME test results of immunochromatographic strips after storage for 3 months at room temperature. The concentrations of AME were 0, 0.1, 1, 5, 10, 20, 30, and 40 ng/mL, respectively.

A batch of assembly completed immunochromatographic strips were prepared at the same time. Some of them (stored 0 days) were used for studying the detection limit (Figure 5). Other immunochromatographic strips were kept at room temperature for 3 months in a zip-lock plastic bag containing silica gel desiccant and were used for evaluating the stability performance. These strips were used for the detecting a series of different concentrations AME of 0, 0.1, 1, 5, 10, 20, 30, 40 ng/mL. The results of Figure 7 showed that the density of the color on T line and C line seem slightly weaker compared to those immunochromatographic strips which were stored 0 days, but the loss of the intensity of red density was not significant. The results showed that the colloidal gold immunochromatographic strip developed in this study has good stability, and it may be suitable as a commercial kit for rapid detection.

### 2.8. Application of the Immunochromatographic Strip to Spiked Fruit Samples

**Figure 8:**
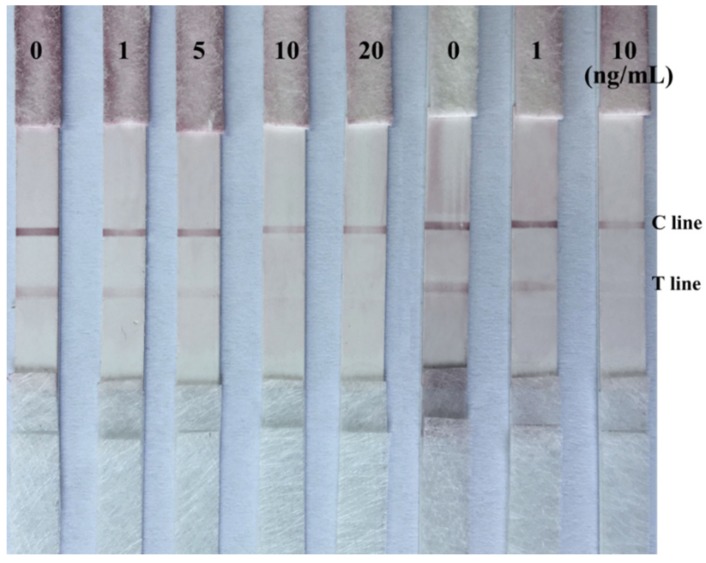
Immunochromatographic strip test of AME in spiked cherry and orange samples. The spiked concentrations of AME in cherry were 0, 1, 5, 10, and 20 ng/mL, and in orange were 0, 1, and 10 ng/mL.

In order to apply the immunochromatographic strip to a real sample matrix, fruit samples of cherries and oranges spiked with AME standards were determined by the prepared colloidal gold immunochromatographic strip. The recoveries of AME in spiked cherry and orange fruits determined by competitive ELISA were 86.1% and 80.7%, respectively. The AME concentrations of 0, 1, 5, 10, and 20 ng/mL in cherries were prepared by diluting the extracted samples using PBS buffer. Then the extracted cherry samples were detected by the immunochromatographic strip. Additionally, the spiked concentrations of AME in orange (0, 1, and 10 ng/mL) were also detected by the immunochromatographic strip. All of the experiments were repeated three times. The detection results are shown in Figure 8. The density of the red color at T line and C line was similar to the result of Figure 5. When the concentration of AME toxins increased, the intensity of the red color on T lines reduced. The detection limit of the strip for AME was also about 10 ng/mL. All of the results indicated that the real sample matrix has very little impact on the detection result of the strip, and the immunochromatographic strips were reliable for AME detection in fruits.

## 3. Discussion

Alternariol monomethyl ether (AME) toxin is one of the most commonly found *Alternaria* mycotoxins in fruits and their processed products. AME could cause serious diseases in humans and animals because of its mutagenicity, teratogenicity, and carcinogenicity. Although the statutory or guideline limits have not been set for AME toxin in food and agricultural products by the regulatory authorities, recently the European Standing Committee suggested that EU member states should collect the occurrence data of *Alternaria* mycotoxins containing AME toxin in food. In addition, the European Food Safety Authority (EFSA) has published its scientific opinion about the health risk of AME toxin in food and feed [2]. So, the problem of food safety about AME toxin has caused wide public concern over the recent years.

Similar to most mycotoxins, AME is also a low molecular weight nonimmunogenic toxin, so it is necessary to couple AME with carrier proteins for antibody production. Structurally, AME does not have active groups that can be conjugated with the carrier protein bovine serum albumin (BSA). Thus, in this study, a carboxylic group from methyl 4-bromobutanoate was linked to the carbonyl group of AME toxin through derivation, allowing AME toxin to covalently bind with the amino group of BSA carrier protein. NMR and HPLC-MS results showed that the AME linked carboxylic acid was successfully obtained. The conjugation of AME linked carboxylic acid and BSA was prepared through the amide bonds using 1-ethyl-3-(3-dimethylaminopropyl) carbodiimide hydrochloride (EDC) and the dehydrating agent N-hydroxysuccinimide (NHS). AME-BSA was used for immunization, the anti-AME mAb was obtained and displayed a higher affinity for AME, the antibody titer (>1:90,000) was obtained, and the IC_50_ values of AME was 0.227 ng/mL. The selected anti-AME monoclonal antibody showed lower cross-reactivity (2.1%) with the AME analogues AOH. So far, there are still no relevant reports about the preparation and use of the AME monoclonal antibody.

A competitive colloidal gold immunochromatographic strip for the detection of AME in fruit samples has been developed using the selected anti-AME mAb. The CGNs were round in shape and well dispersed. The smaller particle size of CGNs means that they have a larger specific surface, could combine a larger amount of antibodies, and have good stability, but had a lighter red color on T line, and vice versa. The average diameter of CGNs was about 24 nm, which was widely used in previous literature [22,26]. The naked CGNs had a UV absorption wavelength at 517 nm. When it was covered with anti-AME mAb by strong electrostatic interaction, the UV absorption wavelength experienced a red-shift [27]. Here, the UV absorption wavelength of colloidal gold-mAb conjugates was 524 nm. The intensity of red density of the test line (T line) was inversely proportional to AME concentration in the range 0.1–10 ng/mL. The visual limit of detection (LOD) of AME was found to be about 10 ng/mL. Recently, the detection of some other mycotoxins also used the same typical method, and similar results were also obtained. For example, the detection limits of 50, 5, 40, and 20 ng/mL were obtained with the naked eyes for deoxynivalenol [18], ochratoxin A [15], T-2 [25], and zearalenone [22,26], respectively. The prepared immunochromatographic strips showed no significant cross-reactivity with AOH, ALT, and TeA which have a structure similar to AME. The results indicated that the presence of these mycotoxins in the test sample did not interfere with the detection of AME. In addition, the strips could be used for 3 months without significant loss of activity. All of the results showed that the prepared immunochromatographic strips have good specificity and stability.

Finally, the prepared colloidal gold immunochromatographic strips were used for the detection of the cherry and orange samples spiked with AME standards. The results showed that the real sample matrix has very little impact on the detection result of the strips. All of the results indicated that the proposed competitive colloidal gold immunochromatographic strips developed in this work are suited for real-time, rapid, or portable semi-quantitative detection of AME toxin in fruits. Moreover, it may be suitable as a commercial kit for AME detection. Furthermore, the antibodies of other main *Alternaria* mycotoxins (such as AOH, TeA, etc.) were also produced. Then the immunochromatographic strips were prepared using the antibodies for the simultaneous, rapid, portable, and visual detection of multiple *Alternaria* mycotoxins.

## 4. Materials and Methods

### 4.1. Materials

Alternariol (AOH), alternariol monomethyl ether (AME), altenuene (ALT), tenuazonic acid (TeA), *N, N*-dicyclohexylcarbodiimide (DCC), *N*-hydroxysuccinimide (NHS), and gold chloride (HAuCl_4_·3H_2_O) were purchased from Sigma-Aldrich (Shanghai, China). Bovine serum albumin (BSA), human serum albumin (HSA), methyl 4-bromobutanoate, trisodium citrate dihydrate, and Tween-20 were purchased from Amresco (CochranSolon, OH, USA). PEG20000 was purchased from Alfa Aesar Chemical Co., Ltd. (Shanghai, China). Acetonitrile (HPLC grade) were purchased from Thermo Fisher Scientific (Waltham, NJ, USA). Incomplete Freund’s adjuvant, complete Freund’s adjuvant, 96-well microplates, goat anti-mouse IgG, and developing solution was offered by Beijing Kwinbon Biotechnology Co., Ltd. (Beijing, China). Polyvinyl chloride (PVC) sheet, NC membrane (Millipore 135), absorbent pad, conjugate pad, and sample pad were obtained from Shanghai Kinbio Tech. Co., Ltd. (Shanghai, China). Analytical grade sodium chloride (NaCl), dimethylformamide (DMF), methyl 4-bromobutanoate, potassium carbonate (K_2_CO_3_), ethanol, petroleum ether, tetrahydrofuran (THF), lithium hydroxide (LiOH), ethyl acetate (EtOAc), and other chemicals and solvents were ordered from Beijing Chemical Reagent Co. (Beijing, China). Water was deionized and purified with a Milli-Q system (Millipore, Bedford, MA, USA).

### 4.2. Carboxyl Derivative Modification of AME

AME derivative with carboxyl group was synthesized as follows. Methyl 4-bromobutanoate (145 mg, 0.80 mmol) and K_2_CO_3_ (302 mg, 2.19 mmol) were added to a solution of AME (200 mg, 0.73 mmol) in DMF (10 mL). The resulting solution was stirred at 50 °C for 2 h. Then the mixture was cooled, diluted with H_2_O (20 mL), and extracted with ethanol (3 × 20 mL). The organic phase was combined, dried, and concentrated. The residue was purified by column chromatography on silica gel (petroleum ether:ethanol = 2:1) to give AME linked methyl ester (compound **1**) as a light yellow solid (205 mg).

H_2_O (1 mL) was added to a solution of compound **1** (150 mg, 0.40 mmol) in CH_3_OH (5 mL), THF (5 mL), LiOH (18 mg, 1.20 mmol). The resulting solution was stirred at room temperature for 5 h. The solvent was concentrated and the residue was diluted with H_2_O (5 mL), adjusted to pH = 4, and then extracted with ethyl acetate (EtOAc) (3 × 10 mL). The organic phase was combined, dried and concentrated, and recrystallized by EtOAc to give AME linked carboxylic acid (compound **2**) as a white solid (40 mg).

### 4.3. Preparation of AME-BSA and AME-OVA

AME-BSA was prepared according to the following steps. DCC (13.7 mg) was added was added to a solution of AME linked carboxylic acid (15.8 mg) in DMF (1.5 mL). The mixture was stirred for 30 min, and then added with NHS (7.6 mg). The solution was stirred at room temperature under dark conditions for 3.5 h. The precipitation was removed by centrifugation. The supernatant was added dropwise into the bovine serum albumin (BSA) solution (0.1 M), which was stirred at 4 °C under dark conditions for 2 h. In addition, to prepare AME-OVA, AME linked carboxylic acid (9.2 mg) and EDC (7.4 mg) were added to DMF (2 mL), and stirred for 20 min. The resulting solution was added dropwise into the ovalbumin (OVA) solution (10 mg/mL), which was stirred at 4 °C under dark conditions for 24 h. Finally, the solution of AME-BSA and AME-OVA were dialyzed using PBS buffer for 72 h, with three changes of PBS every 24 h.

### 4.4. Preparation of Anti-AME mAb

The Balb/c mouse was immunized with 200 μg of AME-BSA in PBS buffer, which was mixed with comparable amount of complete Freund’s adjuvant. From the second immunity to the fourth immunity, the complete Freund’s adjuvant was replaced by incomplete Freund’s adjuvant. The interval time of each immunity was 15 days. When the titer of serum was relatively high, the booster immunization was implemented using 200 μg AME-BSA. After 15 days, spleen cells of the immunized mice and SP2/0 cells were fused, and the positive cell clones were screened using limited dilution method. The ascites monoclonal antibodies (mAbs) were prepared using the conventional technique, and purified by saturated ammonium sulfate.

### 4.5. Competitive ELISA

A 96-well microplate was coated with AME-OVA for 2 h at 37 °C, and then was placed overnight at 4 °C. AME, diluted in PBS at 0, 0.01, 0.1, 0.3, 0.9, 2.7, and 8.1 ng/mL, was mixed with equal volume anti-AME mAb (90,000-fold diluted in PBS) for 30 min at 37 °C, respectively. The resulting solution was added to the 96-well microplate (100 μL/well), and incubated for 30 min at 37 °C. Following a washing step, goat anti-mouse IgG was added (50 μL/well). Developing solution (100 μL/well) was added and incubated for 15 min at 37 °C, and then was stopped with 2 M HCl (50 μL/well). The optical densities (ODs) were measured at 450 nm using a microplate reader.

### 4.6. Preparation of Colloidal Gold Nanoparticles (CGNs) and CGNs-mAb Conjugates

Colloidal gold nanoparticles (CGNs) and the colloidal gold-mAb conjugates were prepared according to the previous approach with minor modification [28]. Monoclonal antibodies concentration and pH for conjugation of mAbs with CGNs were optimized (see Supplementary Material). Briefly, 0.01% HAuCl_4_ solution (100 mL) was boiled, and 1% trisodium citrate solution (1.6 mL) was subsequently and rapidly added into the boiling solution. Then the solution was boiled for 10 min and cooled down to room temperature. The conjugation conditions between CGNs and anti-AME mAb, including the mAb concentration and the pH, were optimized before conjugation. The results showed that the optimal mAbs concentration was 7.2 μg/mL and the optimal pH value of colloidal gold was 8.0. The conjugation of colloidal gold with anti-AME mAb was conducted according to the following method. Briefly, 5 mL of CGNs was mixed with 40 μg of mAbs, incubated for 2 h at room temperature, and then was blocked by BSA solution. After incubation, colloidal gold-mAb conjugates were purified and concentrated by centrifugation. Finally, the colloidal gold-mAb conjugates were resuspended in 0.5 mL of Tris-HCl buffer (10 mM) containing 5% sucrose, 1% BSA, 0.1% Tween-20, and 0.1% PEG 20000.

### 4.7. Preparation of Colloidal Gold Immunochromatographic Strip

The colloidal gold immunochromatographic strips were prepared as previously described [13,19]. The immunochromatographic strips were assembled by the sample pad, conjugate pad, nitrocellulose (NC) membrane, absorption pad, and polyvinyl chloride (PVC) soleplate. A total of 1 μL per 1 cm of AME-OVA conjugates (2 mg/mL) and goat anti-mouse IgG antibody (1 mg/mL) was sprayed onto NC membrane as the test line (T line) and control line (C line), respectively, and then dried at 37 °C for 2 h. The distance between the T line and the C line was 5 mm. The whole assembled sheet was cut into strips (5 mm × 60 mm). The strips were stored under dry conditions for further use at room temperature.

### 4.8. Samples and Preparation

The sample was extracted according to Wang et al. [29]. Orange and cherry fruits were purchased from local markets. One kilogram of orange and cherry were cut into small pieces, and then comminuted to a mash by a homogenizer. The homogenized cherry (5 g) and orange (5 g) were placed into 50 mL centrifugation tubes, and added with water to 5 mL. Subsequently, 2 μL of AME standard sample (1 mg/mL) and 20 mL of the acetonitrile containing citric acid (100 mM) were added. The centrifugation tubes were shaken for 30 min at 150 rpm. Then NaCl (2 g) was added and centrifuged at 10000 rpm for 5 min. The upper acetonitrile layer was purified by the SPE cartridge. The extract of 4 mL was dried under nitrogen, and reconstituted with 0.1 mL acetonitrile. Finally, the reconstituted solution was diluted using 0.4 mL PBS buffer. The recoveries of AME in cherry and orange were detected by competitive ELISA, respectively.

## Supplementary Materials

The following are available online at www.mdpi.com/2072-6651/9/5/152/s1, Figure S1: Determination of the optimal monoclonal antibodies concentration by visual observation, Table S1: The optimal monoclonal antibodies concentration by visual observation, Figure S2: The optimal pH value determination of colloidal gold by visual observation, Table S2: The optimal pH value determination of colloidal gold by visual inspection.
