# Supplementary material for: Development of an Immunochromatographic Strip Test for the Rapid Detection of Alternariol Monomethyl Ether in Fruit
Source: Toxins (Basel). 2017 Apr 29;9(5):152. doi: 10.3390/toxins9050152 (PMC5450700; doi:10.3390/toxins9050152)
Supplement: Supplementary file 1 [file toxins-09-00152-s001.pdf]

Supplementary Materials: Development of an Immunochromatographic Strip Test for the Rapid Detection of Alternariol Monomethyl Ether in Fruit

Yan Man, Gang Liang, Fuchao Jia, An Li, Hailong Fu, Meng Wang and Ligang Pan

Optimal monoclonal antibodies concentration for conjugation with colloidal gold nanoparticles.

Table S1. The optimal monoclonal antibodies concentration by visual observation.

Number	Colloidal Gold (μL)	Anti-AME mAb (μg)	Tris-HCl (pH 8.0) (μL)	10% NaCl (μL)	Color
1	1000	0	100	100	blue-black
2	1000	0.5	99	100	blue-black
3	1000	1	98	100	blue-black
4	1000	2	96	100	blue-violet
5	1000	4	92	100	purplish red
**6**	**1000**	**6 ***	**88**	**100**	**red**
7	1000	8	84	100	red
8	1000	10	80	100	red
9	1000	12	76	100	red

* Shows optimal monoclonal antibodies concentration.

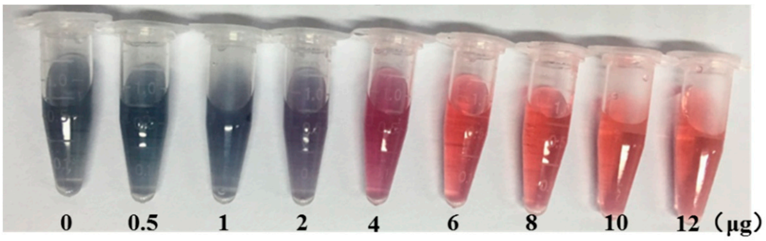

Figure S1. Determination of the optimal monoclonal antibodies concentration by visual observation. Six micrograms of monoclonal antibodies per 1 mL of colloidal gold was chosen as the minimal amount. The optimal concentration monoclonal antibodies was 7.8 μg/mL colloidal gold (i.e, 20 % excess of the minimal amount).

Table S2. The optimal pH value determination of colloidal gold by visual inspection.

Number	Colloidal Gold (μL)	Anti-AME mAb (μg)	pH Vaules	10%NaCl (μL)	Color
1	500	3	6.5	50	red
2	500	3	7.0	50	red
3	500	3	7.5	50	red
**4**	**500**	**3**	**8.0 ***	50	red
5	500	3	8.5	50	purplish red
6	500	3	9.0	50	purplish red
7	500	3	9.5	50	purplish red
8	500	3	10	50	purplish red

* Shows optimal pH value of colloidal gold.

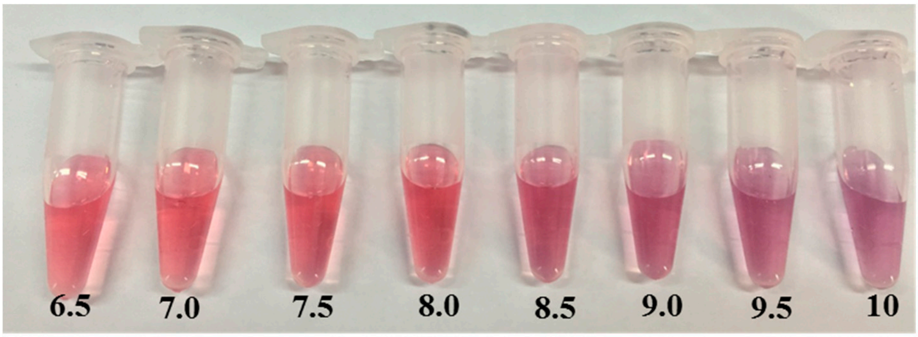

Figure S2. The optimal pH value determination of colloidal gold by visual observation. A pH of 8.0 was the optimal value for conjugation of monoclonal antibodies with colloidal gold nanoparticles.
